# A Novel Mechanism Controlling Resetting Speed of the Circadian Clock to Environmental Stimuli

**DOI:** 10.1016/j.cub.2014.02.027

**Published:** 2014-03-31

**Authors:** Violetta Pilorz, Peter S. Cunningham, Anthony Jackson, Alexander C. West, Travis T. Wager, Andrew S.I. Loudon, David A. Bechtold

**Affiliations:** 1 Faculty of Life Sciences, University of Manchester, Manchester M13 9PT, UK; 2 Pfizer Global Research and Development, Groton, CT 06340, USA

## Abstract

Many aspects of mammalian physiology are driven through the coordinated action of internal circadian clocks. Clock speed (period) and phase (temporal alignment) are fundamental to an organism’s ability to synchronize with its environment. In humans, lifestyles that disturb these clocks, such as shift work, increase the incidence of diseases such as cancer and diabetes. Casein kinases 1δ and ε are closely related clock components implicated in period determination. However, CK1δ is so dominant in this regard that it remains unclear what function CK1ε normally serves. Here, we reveal that CK1ε dictates how rapidly the clock is reset by environmental stimuli. Genetic disruption of *CK1ε* in mice enhances phase resetting of behavioral rhythms to acute light pulses and shifts in light cycle. This impact of CK1ε targeting is recapitulated in isolated brain suprachiasmatic nucleus and peripheral (lung) clocks during NMDA- or temperature-induced phase shift in association with altered PERIOD (PER) protein dynamics. Importantly, accelerated re-entrainment of the circadian system in vivo and in vitro can be achieved in wild-type animals through pharmacological inhibition of CK1ε. These studies therefore reveal a role for CK1ε in stabilizing the circadian clock against phase shift and highlight it as a novel target for minimizing physiological disturbance in shift workers.

## Highlights

• *CK1ε*^−/−^ mice exhibit enhanced phase resetting to advancing and delaying stimuli
• Selective pharmacological inhibition of CK1ε with PF4800567 enhances resetting
• Accelerated resetting is observed in central and peripheral clocks
• Accelerated resetting reduces physiological desynchrony

## Teaser

Pilorz et al. reveal a new role for CK1ε in stabilizing the phase of the circadian clock. Using genetic and pharmacological targeting, they demonstrate that CK1ε dictates how rapidly the clock is reset by photic (light) and nonphotic (temperature) stimuli and thus highlight a novel target for reducing physiological disturbance in shift work.

## Results and Discussion

### Accelerated Phase Resetting to Light in Mice Lacking *CK1ε*

Casein kinase 1 (CK1)-mediated phosphorylation and degradation of PERIOD (PER) protein are crucial determinants of the pace of the circadian clock, and we and others have shown that CK1δ is the principal isoform in regulating clock speed [1–4]. In contrast, genetic deletion or pharmacological inhibition of CK1ε has little impact on circadian period in mice ([5]; see Figure S1A available online), despite the two isoforms being closely related. This lack of effect is not because CK1ε does not engage the clockwork; in its gain-of-function *tau* mutant form, *CK1ε*^*tau*^ dramatically accelerates circadian timing [5]. It thus remains unclear what role CK1ε normally plays in the molecular clockwork.

**Figure 1:**
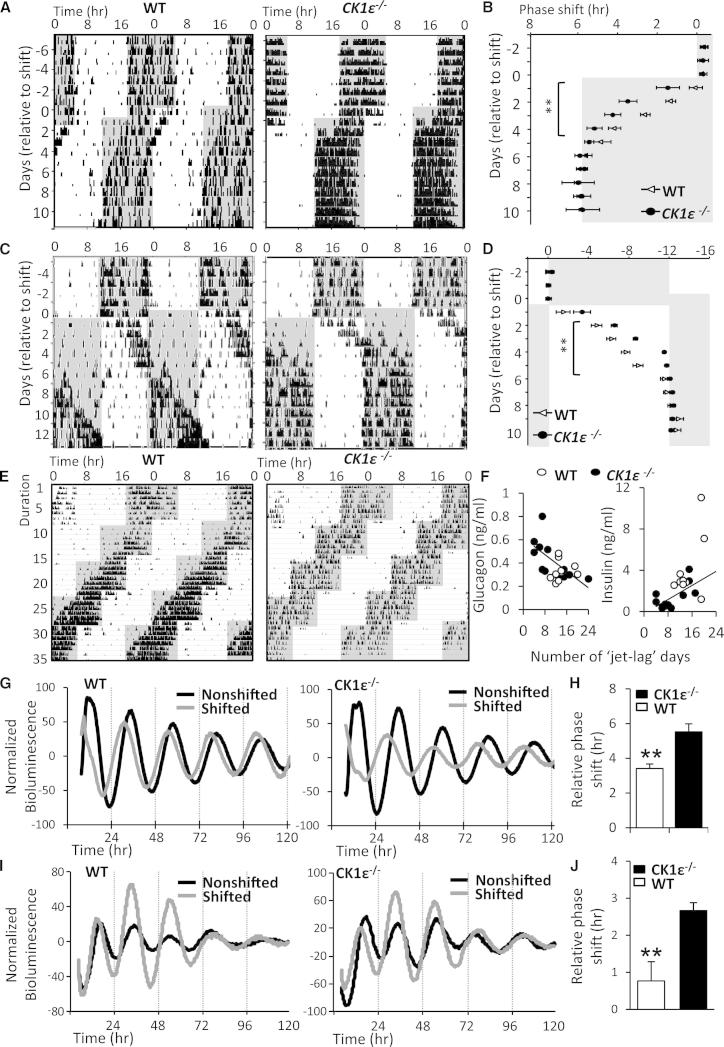
Rapid Entrainment of Behavioral and Physiological Rhythms to Single or Repeated Phase Shifts in *CK1ε*^−/−^ Mice (A–D) WT and *CK1ε*^−/−^ mice were entrained to a 12 hr/12 hr light-dark (LD) cycle for >7 days and then subjected to a 6 hr advance (A and B; n = 13–17/genotype) or 12 hr delay (C and D; n = 8–10/genotype) of the LD cycle. As shown by representative actogram records of wheel-running locomotor activity (A and C) or group analysis of activity onset (B and D), *CK1ε*^−/−^ mice entrained to the new LD phase significantly faster than WT mice. Shading indicates lights-off. Data shown are mean ± SEM; ^∗∗^p < 0.01, repeated-measures two-way ANOVA. (E and F) WT and *CK1ε*^−/−^ mice were subject to four consecutive 6 hr advancing shifts in LD cycle, each separated by 7 days (n = 10–12/genotype). As shown in representative actogram records of wheel-running activity (E), *CK1ε*^−/−^ mice showed accelerated entrainment to each shift in environmental LD cycle. Analysis of serum collected 7 days after the final shift revealed that levels of circulating glucagon and insulin exhibited significant negative and positive correlations, respectively, with severity of jet lag (“jet-lag day” defined as >1 hr phase misalignment between activity onset and lights-off) in the *CK1ε*^−/−^ mice (F; glucagon: r = −0.66, p < 0.05; insulin: r = 0.69, p < 0.05, Pearson correlation). (G–J) The relative phasing of suprachiasmatic nucleus (SCN) and liver clocks in response to a 6 hr advancing phase shift was assessed in WT and *CK1ε*^−/−^ mice using ex vivo analysis of mPER2::Luc bioluminescence recording (n = 5–7 mice/group). SCN slices collected at ZT6 2 days after in vivo phase shift exhibited an advanced phase compared with nonshifted controls (G); this phase advancement was significantly greater in *CK1ε*^−/−^ mice (H). A significantly greater advancement in phase was also observed in liver slices collected from *CK1ε*^−/−^ mice compared with WT animals (I and J). Histograms reflect mean ± SEM; ^∗∗^p < 0.01, ^∗^p < 0.05, t test.

To address this, we first compared the response of wild-type (WT) and *CK1ε*^−/−^ mice to single or repeated advancing (6 hr) or delaying (12 hr) shifts in the timing of environmental light-dark (LD) cycles. Both genotypes showed normal entrainment to a 12:12 hr LD cycle, and progressive adaptation of activity rhythms in response to a shift in LD cycle (Figure 1). However, re-entrainment was accelerated significantly in *CK1ε*^−/−^ mice compared with WT animals. In response to a 6 hr advance, *CK1ε*^−/−^ mice entrained ∼2 days faster than WT mice (WT, 5.8 ± 0.3 days; *CK1ε*^−/−^, 4.1 ± 0.4 days; p < 0.05, U test; n = 13–17/group) (Figures 1A and 1B). Similar rapid re-entrainment of wheel-running activity was observed in the *CK1ε*^−/−^ mice in response to a 12 hr reversal of the LD cycle (WT, 6.8 ± 0.4 days; *CK1ε*^−/−^, 4.4 ± 0.3 days; p < 0.05, U test; n = 8–10/group) (Figures 1C and 1D). In independent experiments, accelerated adaption of *CK1ε*^−/−^ mice to advancing and delaying shifts was also observed in rhythms of body temperature (T_b_), non-wheel-running locomotor activity, and oxygen consumption (Figures S1B–S1D). Desynchrony among internal tissue clocks (and the downstream physiological rhythms they drive) is evident during re-entrainment, as different peripheral tissues exhibit different rates of resetting [6, 7]. By directly comparing rhythms in T_b_ and activity in individual mice subjected to LD phase reversal, we observed desynchronization between the two physiological rhythms (Figure S1E); however, this state of desynchrony was greatly reduced in *CK1ε*^−/−^ mice (3.3 ± 0.5 days, with >1 hr phase dissociation between onset of activity and T_b_ profiles) compared with WT mice (5.5 ± 0.6 days; p < 0.05, U test).

WT and *CK1ε*^−/−^ mice were next subjected to four consecutive 6 hr advancing shifts, each separated by 7 days (Figure 1E; n = 10 or 11/group). As observed with an acute phase shift, *CK1ε*^−/−^ mice were able to repeatedly re-entrain to each new light cycle faster than WT mice, such that the total number of jet-lagged days (defined as >1 hr phase misalignment between activity onset and lights-off) over 4 weeks was significantly reduced in the *CK1ε*^−/−^ mice (WT, 14.3 ± 1.0 days; knockout [KO], 8.7 ± 1.7 days; p < 0.01, U test). Interestingly, levels of circulating glucagon and insulin exhibited significant negative and positive correlations, respectively, with the severity of jet lag in *CK1ε*^−/−^ mice (Figure 1F; glucagon: r = −0.66, p < 0.05; insulin: r = 0.69, p < 0.05, Pearson correlation), and WT mice that exhibited particularly slow phase adaption exhibited greatly elevated circulating insulin (Figure 1H). No genotype differences were observed in these hormones in nonshifted mice (insulin: WT, 2.1 ± 0.4 ng/ml; *CK1ε*^−/−^, 2.5 ± 0.4 ng/ml; glucagon: WT, 0.4 ± 0.1 ng/ml; *CK1ε*^−/−^, 0.5 ± 0.1 ng/ml; Figure S2A). Circadian disruption caused by constant light exposure has been shown to increase weight gain and decrease insulin sensitivity in mice [8], and in humans, chronic shift work is associated with an increased incidence and severity of clinical pathologies, including diabetes [9–11]. Combined with the observation of reduced desynchronization between rhythms in T_b_ and locomotor activity rhythms (Figure S1E), and accelerated restoration of normal rhythms in metabolic rate following acute phase shift (Figure S1B), these results suggest that rapid re-entrainment (or at least that achieved through *CK1ε* targeting) is beneficial for maintaining metabolic homeostasis during a phase shift.

We next assessed whether accelerated re-entrainment of *CK1ε*^−/−^ mice to shifts in the LD environment reflected an enhanced phase adaption of the suprachiasmatic nucleus (SCN) and peripheral clockworks to the new LD cycle. To facilitate these studies, we bred WT and *CK1ε*^−/−^ mice to the mPER2::Luciferase (Luc) reporter mice [12]. Mice were subjected to a 6 hr advance in the LD cycle; SCN and liver slices were then collected for in vitro mPER2::Luc recording on the second day postshift (Figures 1G and 1H). Both genotypes exhibited a significant phase shift in SCN rhythms compared with slices collected from a matched group of nonshifted mice. However, the magnitude of phase advance was significantly greater in the SCN of *CK1ε*^−/−^ mice (Figures 1G and 1H). A significantly greater phase advance was also observed in liver slices derived from *CK1ε*^−/−^ mice (Figures 1I and 1J), suggesting that CK1ε impacts phase resetting of both central and peripheral clocks.

Circadian clock-independent suppression of locomotor activity by light can also accelerate re-entrainment [13]. However, WT and *CK1ε*^−/−^ mice did not differ in their entrainment to long-day (18:6 hr) LD cycles, split (6:6 hr) LD cycles, or short-frequency (11:11 hr and 10:10 hr) LD cycles (Figure S2), demonstrating that neither altered light-induced masking nor aberrant limits of entrainment contribute to the accelerated resetting of *CK1ε*^−/−^ mice. A heightened response to phase-shifting stimuli can be characteristic of low-amplitude oscillators [14]; however, as demonstrated here and in previous studies [5], *CK1ε*^−/−^ mice do not exhibit reduced amplitude rhythms in behavior or SCN oscillation.

### *CK1ε*^−/−^ Mice Exhibit Enhanced Phase Shift in Response to Light

**Figure 2:**
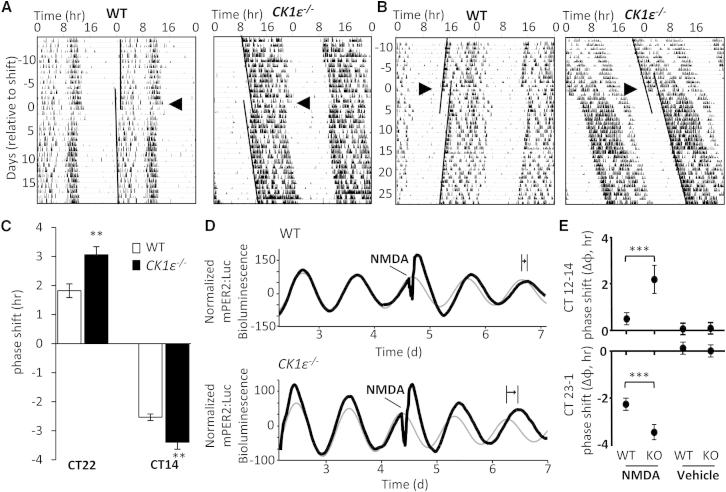
Enhanced Acute Phase Shift Response in *CK1ε*^−/−^ Mice In Vivo and *CK1ε*^−/−^*-*Derived SCN In Vitro (A–C) WT and *CK1ε*^−/−^ mice were maintained in constant darkness (DD) for ∼2 weeks (free-running periods: WT, 24.1 ± 0.02 hr; *CK1ε*^−/−^, 24.3 ± 0.09 hr) and then exposed to light for 1 hr at CT22 (advancing pulse; A) or CT14 (delaying pulse; B). As shown in representative double-plotted actogram records of wheel-running activity (A and B) and analysis of mean phase shift responses (C; n = 10/group), *CK1ε*^−/−^ mice exhibit significantly greater shifts in activity onset compared with WT mice, irrespective of the timing of the light pulse. Histograms reflect mean ± SEM; ^∗∗∗^p < 0.001, t test. Arrowhead represents day of light pulse; black lines on the actograms highlight the phase of activity onset pre and post light pulse. (D and E) To mimic photic input into the SCN in vitro, we kept SCN slice cultures collected from WT and *CK1ε*^−/−^ mice under constant temperature of 37°C and treated them with NMDA (30 μM, 30 min) at either the rise or decline of mPER2:Luc oscillations. Phase delays (or advances) in response to rise (or decline, respectively) in NMDA treatment were measured as the phase difference in the expected (thin gray waveform in D) and observed posttreatment peak of PER2 (thick black oscillation in D). *CK1ε*^−/−^-derived SCN exhibited greater shifts in mPER2:Luc rhythm in response to NMDA treatment compared with WT slices, irrespective of the direction of shift (E). ^∗∗∗^p < 0.001, two-way ANOVA with Tukey’s post hoc test.

The accelerated adaptation to shifts in LD cycle suggested an alteration in the phase-resetting effect of light in the SCN of *CK1ε*^−/−^ mice. To test this, we housed WT and *CK1ε*^−/−^ mice in constant darkness (DD) for >14 days prior to providing an advancing (circadian time [CT] 22) or delaying (CT14) light pulse (Figure 2; n = 13–16/group). In line with the accelerated re-entrainment in *CK1ε*^−/−^ mice, light-induced phase shifts were significantly greater (∼30%) in *CK1ε*^−/−^ mice than those observed in WT mice in response to either an advancing or delaying light pulse (Figures 2A–2C). These findings contrast previous observations made using a different line of *CK1ε*^−/−^ mice [3]; however, mice in that study were initially maintained in DD for an extended period (>3 months) and subjected to repeated pulsing, which may have minimized genotype differences.

To determine whether increased phase resetting to light in *CK1ε*^−/−^ mice reflected an altered response within the SCN itself, we examined clock resetting in SCN slice cultures in response to the neurotransmitter analog N-methyl-D aspartate (NMDA). Retinal inputs to the SCN are principally glutamatergic, and application of NMDA to the SCN in vivo or in vitro can mimic the resetting effects of light [15, 16]. Although application of NMDA timed for the rise or decline of PER2 expression induced phase delays or advances, respectively, in the clockwork of both genotypes (Figures 2E and 2F), SCN tissue derived from *CK1ε*^−/−^ mice exhibited significantly greater phase delays (p < 0.05, two-way ANOVA; n = 5/group) and advances (p < 0.05, two-way ANOVA; n = 3–5/group).

Together, these findings demonstrate that loss of CK1ε activity significantly enhances light-induced phase shift of the SCN clockwork and indicate that the enzyme normally buffers the clock against perturbation across multiple phases of the cycle.

### CK1ε Influences Nonphotic Phase Responses of the Clockwork

**Figure 3:**
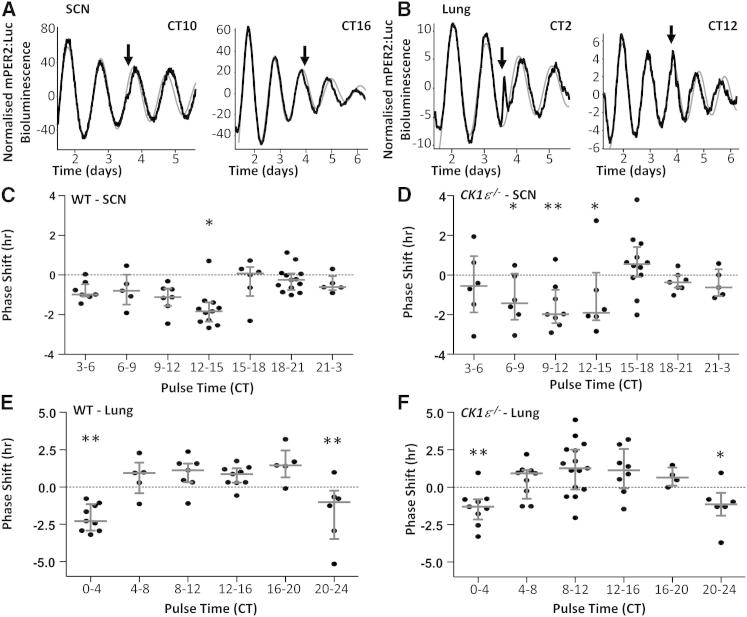
Temperature-Induced Phase Resetting of Central and Peripheral Clocks Temperature pulse-induced phase response curves (PRCs) were generated for WT and *CK1ε*^−/−^-derived SCN (A, C, and D) and lung (B, E, and F) clocks using ex vivo mPER2:Luc bioluminescence recording (n = 42–50 slices/tissue/genotype). A temperature pulse (2 hr, 38.5°C) was applied, and phase shift was calculated as difference between the expected (thin gray waveform) and observed (thick black line) peaks in bioluminescence post temperature pulse (A and B). A clear phase-dependent PRC was observed in both tissues (C and E). Importantly, in comparison to WT-derived tissues, the variability of temperature-induced phase shifts was significantly increased in *CK1ε*^−/−^ SCN (p < 0.05, F test), indicating a role for this enzyme in stabilizing phase responses of the SCN clockwork to nonphotic stimuli. A similar increase in variability was observed in *CK1ε*^−/−^ lung cultures. Data points reflect independent slice cultures shown with median (line) ± interquartile range. ^∗^p < 0.05, ^∗∗^p < 0.01 versus CT15–CT18 in (C) and (D); ^∗^p < 0.05, ^∗∗^p < 0.01 versus CT12–CT16 in (E) and (F); two-way ANOVA with Tukey’s post hoc test.

We next tested whether the altered phase resetting is specific to the effects of light input to the SCN or is a more general feature of the clock. Temperature is a potent entraining stimulus for peripheral tissue clocks [17–19]. Therefore, to explore more directly the impact of CK1ε on circadian clock responses to nonphotic stimuli, entrainment and phase resetting of SCN and lung slice cultures to physiologically relevant temperature cycles (Figure S3) or acute temperature pulses were examined (Figure 3). For cycling studies, SCN and lung slice cultures derived from WT and *CK1ε*^−/−^ mice were maintained for 7 days on 12 hr, 36°C/12 hr, 38.5°C temperature cycles, after which the cycle was reversed and maintained for a further nine cycles. In both genotypes, SCN and lung rhythms of mPER2:Luc bioluminescence entrained rapidly (approximately three cycles) to the reversed temperature cycle, and this new phase was maintained upon release to constant conditions (36°C). These data clearly demonstrate that, in contrast to recent suggestion [17–19], SCN tissue slices are responsive to a temperature-induced phase shift. However, other groups have also reported temperature responses of the SCN [20, 21]. The rapid entrainment of SCN and lung tissues to a reversal of the temperature cycle (2–3 days) makes it difficult to discern whether loss of CK1ε enhanced responses. However, *CK1ε*^−/−^-derived tissues exhibited less disruption in rhythmicity during the temperature reversal and showed an accentuated shift response in the lung clock rhythms compared with WT slices (Figure S3; p < 0.05, repeated-measures two-way ANOVA).

Next, phase-response curves (PRCs) were generated for SCN and lung slice cultures derived from WT or *CK1ε*^−/−^ mice (Figure 3). Tissue slices (n = 42–50 slices/tissue/genotype) were maintained at a constant temperature of 36°C for three cycles prior to receiving a 2 hr ambient temperature pulse of 38.5°C, administered to individual slice cultures at different phases across the circadian cycle. The resulting phase shift was measured as the difference in the observed and predicted peaks of mPER2:Luc bioluminescence (Figures 3A and 3B). A significant phase dependence of temperature-induced shift was observed in WT-derived SCN slices (Figure 3C; CT time of pulse: F(6,91) = 4.3, p < 0.001; genotype: F(1,91) = 0.23, two-way ANOVA) and lung slices (Figure 3E; CT time of pulse: F(6,80) = 13.8, p < 0.001; genotype: F(1,80) = 0.24, two-way ANOVA). The temperature PRC of the SCN generated here is strikingly similar to that previously generated in rat SCN slices using electrical activity recording [20]. Interestingly, the shape of the temperature PRC differed between SCN and lung tissues, such that pulses delivered between CT9 and CT15 elicited a maximal phase advance in SCN slices but induced phase delays in lung. This observation parallels the difference in the direction of phase re-entrainment of the two tissues observed in the temperature cycle reversal experiments (Figure S3). Although the temporal phase dependence of the temperature PRC was similar between genotypes, *CK1ε*^−/−^-derived SCN tissue exhibited significantly greater variability in phase shift responses compared to WT tissue (F = 2.8, p < 0.001, F test). A similar heightened variability in temperature-induced shift was also revealed in *CK1ε*^−/−^-derived lung tissue pulsed between CT4 and CT20 (Figure 3F; F = 2.1, p < 0.05, F test). Taken together, these findings indicate that the influence of CK1ε on phase adaption is a general property of the clockwork, affecting central and peripheral clock responses to photic and nonphotic stimuli.

Previous reports have suggested that CK1ε plays a role in temperature compensation of the molecular clockwork [22, 23]. To ensure that this possibility did not confound our studies, we tested the effect of ambient temperature on periodicity of mPER2:Luc rhythms in SCN and lung slice cultures derived from WT and *CK1ε*^−/−^ mice (Figure S3). No significant differences in the temperature dependence of circadian period were observed between genotypes, and both tissues exhibited robust temperature compensation (SCN Q10: WT, 1.1; *CK1ε*^−/−^, 1.1; lung Q10: WT, 0.9; *CK1ε*^−/−^, 0.9). This suggests that CK1ε is not essential for temperature compensation, likely due to temperature-compensated activity of CK1δ.

### Pharmacological Inhibition of CK1ε Enhances Phase Resetting

**Figure 4:**
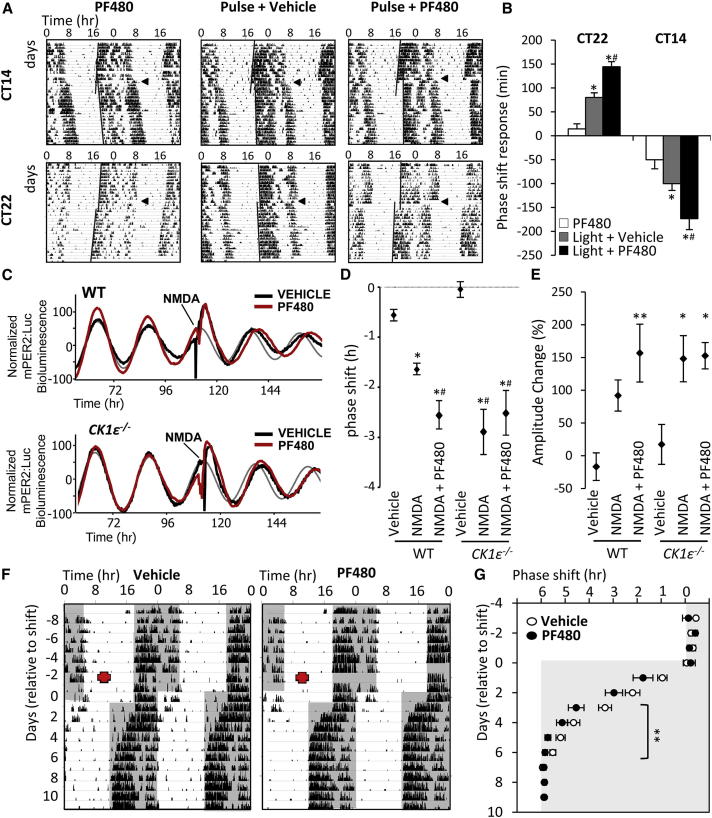
Pharmacological Inhibition of CK1ε Enhances Phase Resetting In Vitro and In Vivo (A and B) WT mice were maintained in DD and then exposed to light for 1 hr at CT14 (delaying pulse; A, top) or CT22 (advancing pulse; A, bottom). Vehicle or CK1ε inhibitor PF4800567 (PF480, 100 mg/kg) was administered as the mice were returned to DD. Another set of mice received PF4800567 at the appropriate times (CT15 and CT23) but were not exposed to a light pulse. As shown in representative double-plotted actogram records of wheel-running activity (A) and analysis of mean phase shift responses (B; n = 6 or 7/group), PF4800567 administration significantly increased light-induced phase shifts. Arrowheads indicate day of pulse; black lines on the actograms highlight the phase of activity onset pre and post light pulse. Histograms reflect mean ± SEM; ^∗^p < 0.01 versus PF4800567 alone; #p < 0.01 versus pulse + vehicle by one-way ANOVA. (C–E) SCN slices cultures collected from WT and *CK1ε*^−/−^ mice were treated with NMDA (30 μM, 30 min; n = 5 or 6/group) in the presence or absence of PF4800567 (1 μM, chronic application from time 0). PF4800567 had no significant effect on period or phase of mPER2:Luc bioluminescence rhythms in either genotype prior to NMDA. However, CK1ε inhibition increased significantly the delaying phase shift in response to peak treatment with NMDA in WT- but not *CK1ε*^−/−^-derived SCN slices (C and D). Phase delays in response to NMDA treatment were measured as the phase difference in the expected (thin gray waveform) and observed posttreatment peak of PER2 (thick black/red oscillations) (C). PF4800567 significantly enhanced the acute induction of PER2::Luc in response to NMDA in WT- but not *CK1ε*^−/−^-derived SCN slices (E). Data reflect individual recordings (C) and group mean ± SEM (D). ^∗^p < 0.01 versus vehicle treatment, #p < 0.01 versus NMDA-treated WT, two-way ANOVA with Tukey’s post hoc test. (F and G) Assessment of pharmacological inhibition of CK1ε on phase resetting in vivo. WT mice were entrained to a 12:12 LD cycle before implantation of a 7-day-release osmotic minipump containing either vehicle or PF4800467 (12.5 μg/hr; n = 14/group). Two days postimplantation, the mice were subjected to a 6 hr phase advance of the LD cycle (F), and daily onset of activity was determined each day (G). Mice treated with PF4800567 exhibited a significant acceleration in the time required to re-entrain locomotor activity rhythms to the new LD cycle. Shading indicates lights-off; red crosses indicate the timing of pump implantation. Results are presented as representative actograms of wheel-running locomotor activity (F) and mean ± SEM for daily phase of activity onset (G). ^∗∗^p < 0.01, repeated-measures two-way ANOVA.

Our results demonstrate a role for CK1ε in stabilizing the circadian clockwork against photic- and nonphotic-induced phase-resetting stimuli. We next tested whether the enhanced phase resetting observed in *CK1ε*^−/−^ mice could be recapitulated in WT mice and tissues by pharmacological inhibition using a highly selective inhibitor of CK1ε, PF4800567 (>20-fold selectivity for CK1ε over CK1δ [1]). Indeed, administration of PF4800567 (100 mg/kg) enhanced the phase shift response of DD-housed WT mice to either an advancing (CT22) or delaying (CT14) light pulse (Figures 4A and 4B). Furthermore, in SCN slice cultures treated with NMDA, application of PF4800567 (1 μM, from time 0) was effective at increasing the magnitude of NMDA-induced phase shift of mPER2:Luc rhythms in WT-derived tissue (Figures 4C and 4D; p < 0.01; n = 5 or 6/group). In contrast, PF4800567 did not alter the magnitude of NMDA-induced phase shift in *CK1ε*^−/−^ tissue. This indicates that the enhanced phase shift in WT SCN is specifically due to targeting of CK1ε.

Phase resetting of behavioral and molecular rhythms by light has been well characterized and in simplified terms reflects an intensity-dependent induction of *Per* expression in the SCN [24, 25]. Inhibition of CK1 activity has been shown to attenuate rates of PER phosphorylation-mediated degradation in SCN and peripheral tissues [1–5, 26]. Consistent with CK1-induced PER2 degradation and enhanced phase shift responses following CK1ε inhibition, the acute induction of mPER2:Luc bioluminescence in WT SCN in response to NMDA application was significantly enhanced by preincubation with PF4800567 (Figure 4E; Figure S4; p < 0.05, two-way ANOVA). Therefore, CK1ε-mediated degradation of PER may normally serve to limit protein accumulation (and resulting phase shifts) in response to light stimulation.

Importantly, pharmacological inhibition in vivo was also effective in accelerating entrainment to shifts in LD cycle. WT mice were implanted with 7-day-release osmotic minipumps containing either vehicle or PF4800567 (12.5 μg/hr). Two days postimplantation, mice were subjected to a 6 hr phase advance of the LD cycle. Mice treated with PF4800567 re-entrained locomotor activity rhythms significantly faster to the new LD cycle (Figures 4F and 4G; vehicle, 5.5 ± 0.4 days; PF4800567, 4.3 ± 0.4 days; p < 0.05, U test; n = 14/group). This ability of pharmacological inhibition of CK1ε to phenocopy the genetic deletion demonstrates that altered shift responses in the KO mice are not due to a developmental impact of CK1ε KO.

### Conclusions

Our results reveal a novel role for CK1ε in resetting of the mammalian circadian clockwork. Targeting of CK1ε by genetic or pharmacological means increased the responsiveness of both central and peripheral clocks to phase-shifting stimuli. This enhanced phase shift response and/or entrainment was demonstrated in vivo and in vitro, and in response in both photic (light in vivo, NMDA treatment in vitro) and nonphotic (temperature) paradigms. Our results indicate that a principle role of CK1ε in the clock may be to limit PER protein accumulation in response to resetting stimuli, and in so doing buffer the clockwork against phase shifts. Selective pressure for such a phase-control mechanism can be envisioned, as it would prevent uncoordinated phase change in response to acute fluctuations in the environment (e.g., weather, predation, food availability). In modern society, however, our lifestyle presents a problem, requiring rapid adaptation of behavioral and physiological rhythms to shift-work schedules or long-haul air travel to which we are not genetically preadapted. Based on our results, inhibition of CK1ε appears to render the SCN less resistant to external perturbation, and hence it resets more rapidly to the external environment. Our studies highlight CK1ε as a potential target for alleviating the detrimental effects of chronic shift work and jet lag.

## Experimental Procedures

### Animals

Male WT (mPER2::Luc [12]) and *CK1ε*^−/−^ (*CK1ε*^−/−^mPER2::Luc [5]) mice 10–16 weeks of age were used for all behavioral experiments. Both males and females were used for ex vivo tissue culture experiments. All experiments were conducted under the aegis of the 1986 Home Office Animal Procedures Act (UK) and local ethical review.

### Behavioral Analysis

Wheel-running activity was recorded and analyzed using ClockLab software (Actimetrics). During 6 hr phase advance, the dark phase was shortened by 6 hr, whereas during 12 hr phase delay, the dark phase was extended by 12 hr. To induce phase shift response to acute light pulse, we housed *CK1ε*^−/−^ and WT mice in constant darkness (DD) for ∼14 days. A 1 hr light pulse was administered at CT14 or CT22, followed by 2 weeks of free run in DD. In selected experiments, WT mice were administered PF4800567 (100 mg/kg) or vehicle (20% cyclodextrin) at the end of the light pulse (CT15 or CT23). Phase shift calculations are detailed in Supplemental Experimental Procedures.

### Tissue Collection for Ex Vivo Phase Analysis

WT and *CK1ε*^−/−^ mice were subjected to a 6 hr advance of the LD cycle. On the second day postshift, mice were culled at zeitgeber time (ZT) 6 of the new LD cycle, and SCN and liver slices were collected for mPER2::Luc recording. A parallel set of mice were not subject to the LD shift and were used as “nonshifted controls.” Phase was measured as the time of the first peak in mPER2::Luc activity after 24 hr in culture.

### Repeated Phase Shift and Serum Analysis

Mice were subjected to a 6 hr phase advance every 7 days for 4 weeks. On the seventh day after the final shift, mice were fasted for 4 hr, and trunk blood was collected at ZT6. Serum insulin and glucagon levels were measured using a mouse diabetes Bio-Plex plate (Bio-Rad) following the manufacturer’s instructions.

### Osmotic Pump

After 7 days of acclimation to wheel-running cages, WT mice were implanted with osmotic minipumps (ALZET Osmotic Pumps Alzet 2001) containing either vehicle (20% cyclodextrin) or PF4800567 (delivered at 12.5 μg/hr for ∼7 days). Two days after pump implantation, the LD cycle was advanced by 6 hr.

### Tissue Culture

Bioluminescence recordings were collected using individual photomultiplier tubes or lumicycle recorder housed within a temperature-cycling incubator. Briefly, WT mPER2::Luc- and *CK1ε*^−/−^ *m*PER2::Luc-derived tissue slices were cut and collected as described previously [2]. For NMDA treatments, incubation temperature was held at 37°C throughout the experiment. NMDA (30 μM) [27] was rapidly added to SCN culture dishes at the appropriate CT, and recording medium was replaced30 min later. The application of NMDA occurred shortly before the third peak or third nadir of PER2 to induce advances or delays, respectively. To calculate the magnitude of phase resetting, we overlaid the waveform of the NMDA-treated cycle on that of the projected cycle (generated from the two pretreatment cycles). Selective inhibition of CK1ε was achieved by chronic administration of PF4800567 (1 μM) to the recording media [2]. For generation of temperature PRC, slice cultures were maintained at 36°C for at least three cycles, after which incubator temperature was raised to 38.5°C for 2 hr.

### Statistical Analysis

Results are presented as mean ± SEM. Statistical significance of group comparisons was tested with Student’s t test or Mann-Whitney U test for simple intergenotype differences. Multivariate experiments (e.g., genotype and treatment) were analyzed using two-way ANOVA with Tukey’s post hoc analysis. A repeated-measures adjustment was employed when appropriate (e.g., daily onset profiles). An F test was used to assess the differences in variability to temperature-induced phase shifts between genotypes.

## Supplemental Information

Document S1. Figures S1–S4 and Supplemental Experimental Procedures
