# Supplementary material for: A Novel Mechanism Controlling Resetting Speed of the Circadian Clock to Environmental Stimuli
Source: Curr Biol. 2014 Mar 31;24(7):766–73. doi: 10.1016/j.cub.2014.02.027 (PMC3988889; doi:10.1016/j.cub.2014.02.027)
Supplement: Document S1. Figures S1–S4 and Supplemental Experimental Procedures [file mmc1.pdf]

Current Biology, Volume *24*

Supplemental Information

A Novel Mechanism Controlling Resetting Speed of the Circadian Clock to Environmental Stimuli

Violetta Pilorz, Peter S. Cunningham, Anthony Jackson, Alexander C. West, Travis T. Wager, Andrew S.I. Loudon, and David A. Bechtold

Figure S1

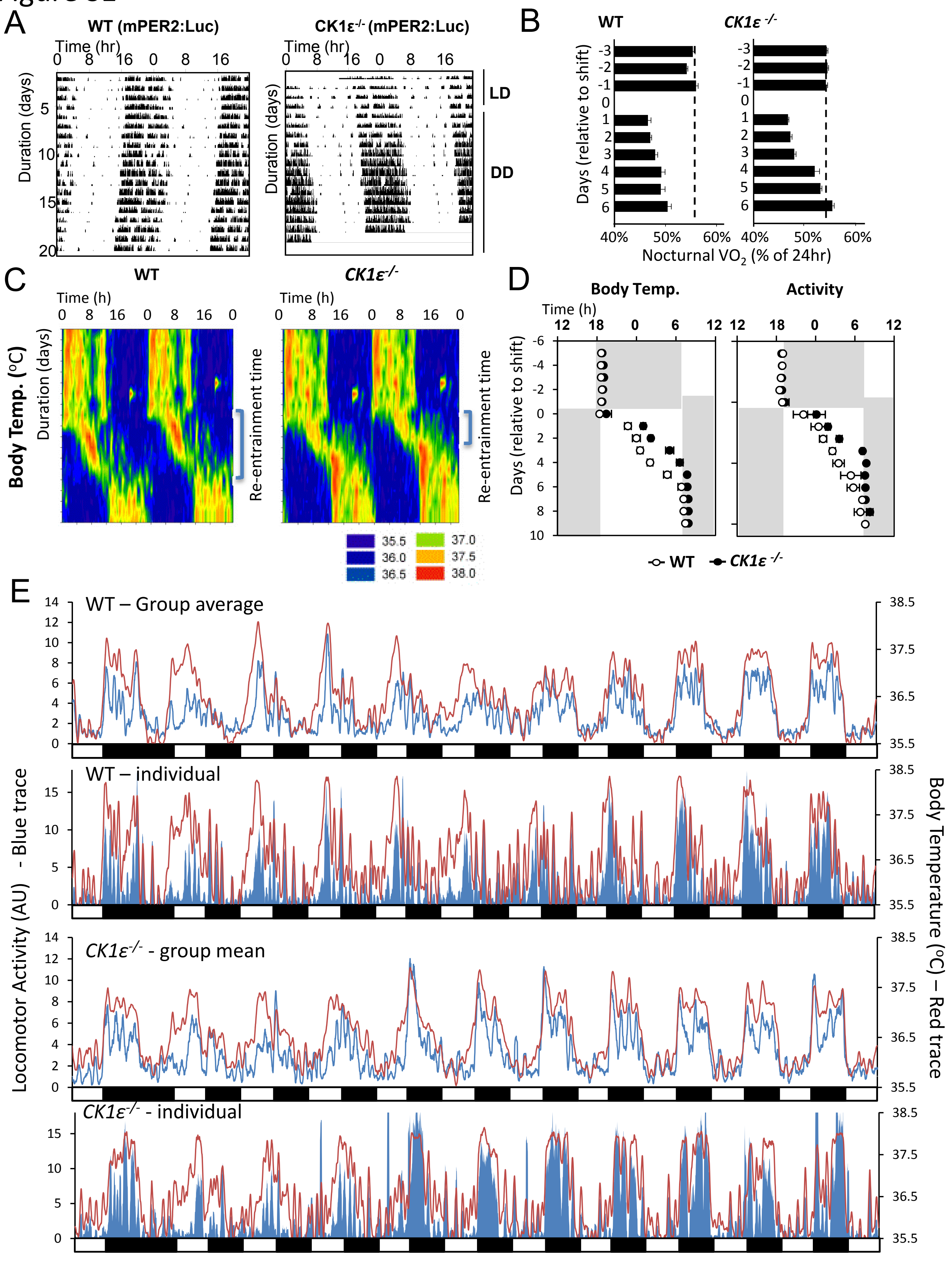

A
WT (mPER2:Luc)
CK1ε-/- (mPER2:Luc)
Time (hr)
Duration (days)
LD
DD
B
WT
CK1ε -/-
Days (relative to shift)
Nocturnal VO2 (% of 24hr)
C
WT
CK1ε-/-
Time (h)
Body Temp. (ºC)
Duration (days)
Re-entrainment time
35.5
36.0
36.5
37.0
37.5
38.0
D
Body Temp.
Activity
Time (h)
Days (relative to shift)
WT
CK1ε -/-
E
WT – Group average
WT – individual
CK1ε-/- - group mean
CK1ε-/- - individual
Locomotor Activity (AU) - Blue trace
Body Temperature (ºC) – Red trace

Figure S2

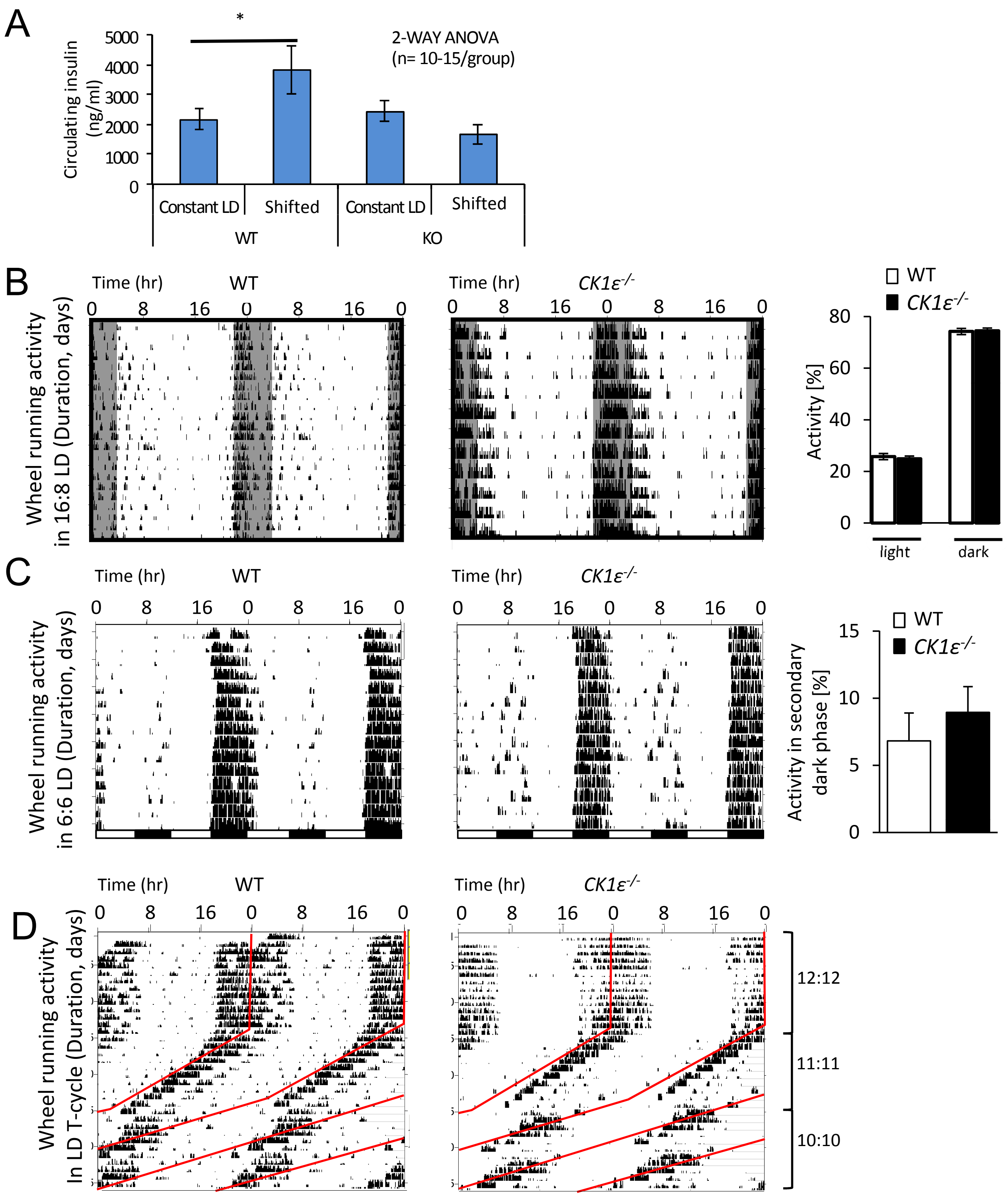

A
*
2-WAY ANOVA
(n= 10-15/group)
Circulating insulin (ng/ml)
5000
4000
3000
2000
1000
0
Constant LD
Shifted
Constant LD
Shifted
WT
KO
B
Wheel running activity in 16:8 LD (Duration, days)
Time (hr)
WT
CK1ε-/-
0 8 16 0 8 16 0
WT
CK1ε-/-
Activity [%]
80
60
40
20
0
light
dark
C
Wheel running activity in 6:6 LD (Duration, days)
Time (hr)
WT
CK1ε-/-
0 8 16 0 8 16 0
Activity in secondary dark phase [%]
15
10
5
0
D
Wheel running activity In LD T-cycle (Duration, days)
Time (hr)
WT
CK1ε-/-
0 8 16 0 8 16 0
12:12
11:11
10:10

Figure S3

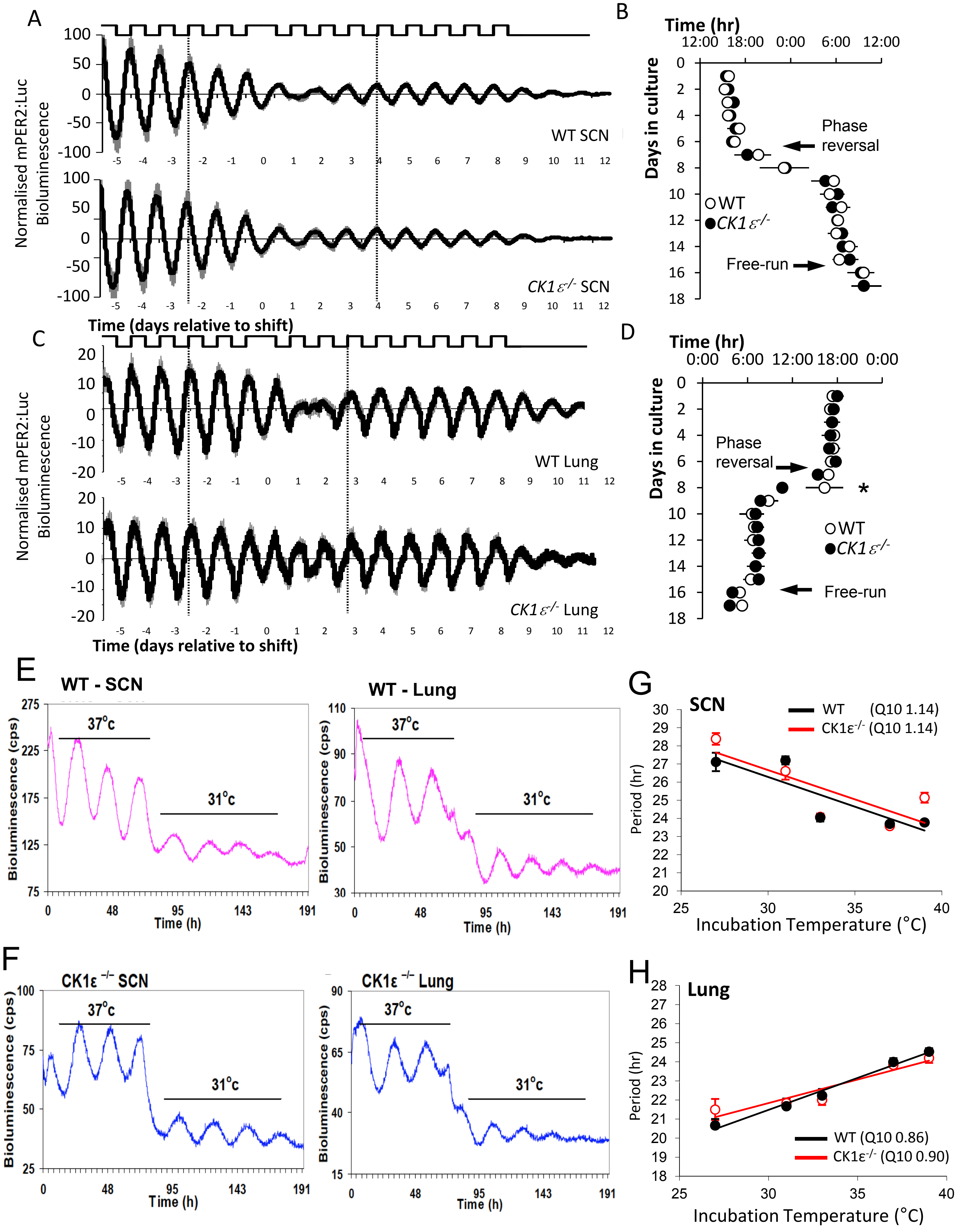
A
Normalised mPER2:Luc Bioluminescence
WT SCN
CK1ε-/- SCN
Time (days relative to shift)
B
Time (hr)
12:00 18:00 0:00 6:00 12:00
Days in culture
Phase reversal
WT
CK1ε-/-
Free-run
C
WT Lung
CK1ε-/- Lung
D
0:00 6:00 12:00 18:00 0:00
*
E
WT - SCN
WT - Lung
37°c
31°c
Bioluminescence (cps)
Time (h)
F
CK1ε -/- SCN
CK1ε -/- Lung
G
SCN
WT (Q10 1.14)
CK1ε-/- (Q10 1.14)
Period (hr)
Incubation Temperature (°C)
H
Lung
WT (Q10 0.86)
CK1ε-/- (Q10 0.90)

Figure S4

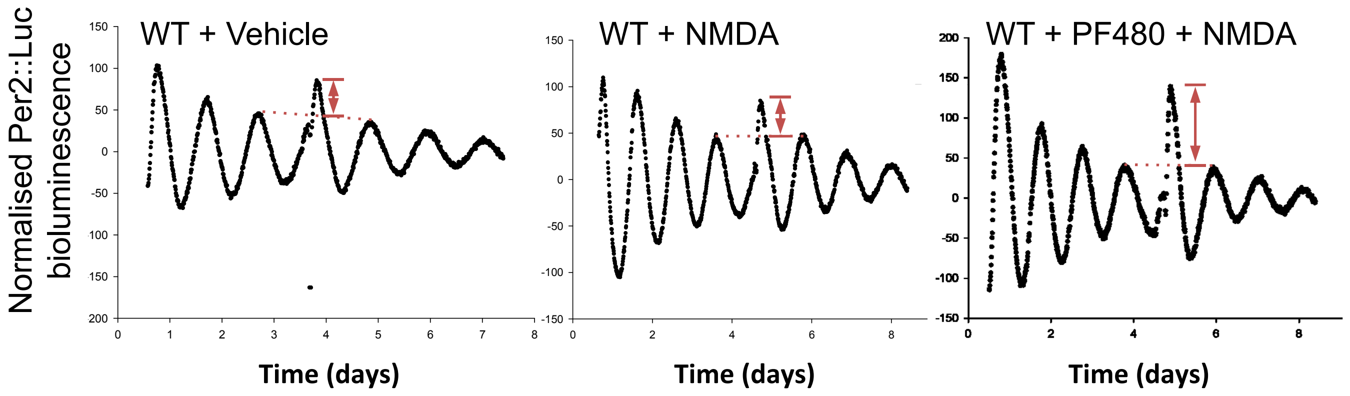
Normalised Per2::Luc bioluminescence
WT + Vehicle
WT + NMDA
WT + PF480 + NMDA
Time (days)
Time (days)
Time (days)

Figure S1. Accelerated entrainment of physiological rhythms to phase shifts in LD cycle in *CK1ε*$^{-/-}$ mice.

A) Representative examples of double plotted actograms of wheel running activity in WT, and *CK1ε*$^{-/-}$ mice under LD and upon release to DD.

In separate experiments where no running wheel was available, an accelerated entrainment to a reversal in LD cycle was also observed in rhythms metabolic gas exchange (**B** VO_2; n=8) and in rhythms of body temperature and locomotor activity (measured by remote telemetry; n=7-8/group; **C-E**) in *CK1ε*$^{-/-}$ mice. **B**) Reversal of the LD cycle disrupted day/night patterns in oxygen consumption in both genotypes. While these were re-established in *CK1ε*$^{-/-}$ mice within 4-5 days, WT mice failed to re-entrain within the duration of the experiment. **C**) Body temperature profiles double plotted in heat-map format, showing the accelerated re-entrainment of *CK1ε*$^{-/-}$ mice compared with WT congenic mice. **D**) Group mean analyses of activity and body temperature profiles showing accelerated re-entrainment in *CK1ε*$^{-/-}$ mice following 12 reversal of LD phase. Plots in **D** reflect the onset of consolidated active/warm period. **E**) Group average and individual profiles of body temperature (red profile, right Y-axis) and locomotor activity (blue profile, left Y-axis) collected simultaneously by remote telemetry in WT and *CK1ε*$^{-/-}$ mice during a reversal of the LD cycle. A pronounced de-synchronisation of body temperature and locomotor activity rhythms were observed as the mice entrained to the new LD cycle; yet this period of desynchronisation was significantly shortened in *CK1ε*$^{-/-}$ mice.

Figure S2. Assessment of locomotor activity entrainment to long-day, 6:6 LD, and LD T-cycles in WT and *CK1ε*$^{-/-}$ mice.

A) WT and *CK1ε*$^{-/-}$ mice were subject to 4 consecutive 6-hr advancing shifts in LD cycle, each separated by 7 days (n=10-12/genotype). Analysis of serum collected 7 days after the final

shift revealed that levels of circulating insulin were significantly elevated in response to shift in the WT, but not *CK1ε*$^{-/-}$ mice. *P<0.05 Two-way ANOVA with Tukey post-hoc test.

B,C) To assess masking effects of light and dark-induced activity responses in the mice, WT and *CK1ε*$^{-/-}$ mice were maintained on a long-day (18hr light, 6hr dark) and split (6hr light/6hr dark) LD cycles. **B**) No difference was observed between the two genotypes under an 18:6hr LD cycle in terms of phase of entrainment or the amount of day-time running wheel activity (n = 8). **C**) Similarly, no genotype differences were observed under split LD cycles of 6hr lights on, 6hr lights off. Both genotypes showed entrainment to a single dark period (n=10/genotype). **D**) To assess the limits of light cycle entrainment in the mice, WT and KO mice were first maintained on a 24hr (12:12) LD cycle for 14 days, after which the LD cycle was reduced to 22hr LD for 10 days, and then reduced to 20hr LD for 10 days (n=10/genotype). Both, WT and *CK1ε*$^{-/-}$ mice exhibited a shortening of circadian periodicity in locomotor activity under 22hr light cycles, although neither were able to fully entrain. Once placed under 20hr LD cycle, both WT and *CK1ε*$^{-/-}$ mice free-ran through the LD cycle, as this frequency fell below the limits of entrainment for both genotypes. Red line indicates middle of dark phase of the LD cycle.

Figure S3. Entrainment of SCN and lung clocks to temperature cycles *ex vivo*.

SCN (**A,B**) and lung (**C,D**) slices were collected from WT and *CK1ε*$^{-/-}$ mice and cultured under ambient temperature cycles of 12hr 36°C/12hr 38.5°C for 7 days, after which the temperature cycle was reversed and maintained for a further 9 cycles before releasing the slices into constant temperature (36°C) conditions. The ambient temperature profile is reflected by the square wave oscillation. The mPER2:Luc oscillations shown in **A** and **C** reflect group average traces (±SEM, shown in grey) of SCN (**A**) and lung (**C**) slices from WT (top) and *CK1ε*$^{-/-}$ (bottom) mice. Vertical dotted lines highlight the phase alignment of mPER2:Luc rhythms with the temperature cycle pre- and post-phase reversal. **B** and **D** illustrate the

phase of the peak of mPER2::Luc bioluminescence across the experiment. WT and *CK1ε$^{-/-}$* derived SCN and lung tissues exhibit rapid re-entrainment to the reversal in temperature cycle. However, *CK1ε$^{-/-}$* SCN and lung tissues exhibited less disruption of PER2 rhythmicity during re-entrainment, and an accelerated phase advance in lung rhythms, in comparison with WT derived tissue.

E,F) To assess the role of CK1ε in temperature compensation of the circadian clockwork, SCN and lung slice cultures were derived from WT and *CK1ε$^{-/-}$* mice (n>4slices/condition/ genotype). Slices were maintained at ambient temperature of 37°C for at least 3 cycles at which point the ambient temperature was raised or lowered (to 29°C, 31°C, 33°C, or 39°C) for a further 4 cycles. The period of mPER2:Luc bioluminescence rhythms were calculated pre- and post-temperature change for SCN (**E**) and lung (**F**). No significant difference was observed in temperature dependency in circadian periodicity or in Q10 values calculated to assess temperature compensation (both SCN and lung produced Q10 values close to 1 which is indicative of strong temperature compensation). A direct effect of temperature on luciferase activity and bioluminescence was clearly observed in temperature compensation studies. However, with lower temperature fluctuation (e.g. between 36.5 and 38C) this direct effect is unlikely to 'mask' genuine mPER2 expression-driven rhythms (**A,B** and Figure 3). Furthermore, the acute affect of 38.5C temperature pulse on mPER2::Luc bioluminescence was tissue dependent (reduced in SCN and increased in lung). **G,H)** both genotypes WT and *CK1ε$^{-/-}$* exhibit similar robust temperature compensation in the SCN as well as in the lung.

Figure S4. CK1ε inhibition increased acute mPER2::Luciferase bioluminescence in response to NMDA in WT SCN slices.

SCN slices cultures collected from WT mice were treated with NMDA (30µM, 30min; n = 5-6/group) in the presence or absence of CK1ε inhibitor PF4800567 (1µM, chronic application

from time 0). CK1ε inhibition increased significantly the amplitude induction in response to peak treatment with NMDA in WT, but not *CK1ε*$^{-/-}$ derived SCN slices.

EXTENDED EXPERIMENTAL PROCEDURES

Housing, light pulse and phase shift analysis: Mice were housed under 12:12hr LD cycle with food and water *ad libitum* unless stated otherwise. Light intensity was ~300 lux for all experiments. For acute light pulse, mice were housed under DD conditions for a minimum of 14 days. Light pulses (1hr) were delivered to individual mice at the appropriate CT time, following which mice were maintained for a further 14 days in DD. We measured light induced phase shifts of free running activity rhythms in DD by using the methods of Daan and Pittendrigh 1976 [S1]. The phase shifts were evaluated by using linear regressions through the activity onsets for more than 10 days before the pulse, and through those for an equivalent interval after re-establishment of a steady-state circadian period. Transitional onsets were not included in the analysis because they represent transient or non steady state cycles of the activity rhythm. For each phase shift we calculated the time difference between the expected time of activity onset and the actual time of activity onset at the first steady state intercept of the post treatment regression line. This method differs from that used by Etchegaray et al [3] during their assessment of phase shift.

In selected experiments, WT mice were administered PF4800567 (100mg/kg) or vehicle (20% cyclodextran) at the end of the light pulse (CT15 or CT23). Non-selective CK1 inhibition has been implicated in NMDA neurotransmission [S2]. Therefore, the timing of administration was delayed to ensure that CK1ε inhibition did not impact on direct responses to light within the retina or retinal-SCN signalling. However, it is possible that direct action of CK1ε at the glutamate receptor contributed to shift responses following NMDA application in vitro.

Repeated phase shift and serum analysis: Mice were subjected to a 6hr phase advance every 7 days for 4 weeks. On the 7th day after the final shift (a time at which all mice had re-

entrained based on running wheel activity), mice were fasted for 4hr and trunk blood collected at ZT6. Serum insulin and glucagon levels were measured using a mouse *diabetes* bioplex plate (BioRad) following manufacturer's instructions. While the animals were not fasted for an extended time (>24hr), removal of food 4hr pre-sampling minimized any effect of acute food intake to alter the endocrine measures in the mice. When samples are collected across the circadian cycle in this manner (4hr fasting relative to collection time) overt rhythms are not observed in either insulin or glucagon. Nonetheless, it is possible that relative phasing of the animals activity contributes to endocrine differences.

S1. Daan, S and Pittendrigh CS. (1976a). A functional analysis of circadian pacemakers in nocturnal rodents. II The variability ofphase response curves. J. Comp. Physiol. 106:253-266.

S2. Chergui, K., Svenningsson, P., and Greengard, P. (2005). Physiological role for casein kinase 1 in glutamatergic synaptic transmission. J Neurosci. *25*, 6601-6609.
